# Multivalent adenoviral vectors which use an antigen capsid-incorporation strategy for HIV vaccination

**DOI:** 10.1186/1742-4690-9-S2-P22

**Published:** 2012-09-13

**Authors:** L Gu, ZC Li, V Krendelchtchikova, A Krendelchtchikov, QL Matthews

**Affiliations:** 1 The Univeristy of Alabama at Birmingham, Birmingham, AL, USA

## Background

Adenoviral (Ad) vectors have been used for a variety of vaccine applications. Traditionally, Ad-based vaccines are designed to express antigens through transgene expression. However, in some cases these conventional Ad-based vaccines have had sub-optimal clinical results. These sub-optimal results are attributed in part to pre-existing Ad serotype 5(Ad5) immunity. To circumvent the need for transgene antigen expression, the “antigen capsid-incorporation” strategy has been developed and used for Ad-based vaccine development. In addition, to increase the magnitude and/or breadth of antigen-specific antibody response, this strategy can be utilized. The major capsid protein hexon has been utilized for antigen display due to hexon’s natural role in the generation of anti-Ad immune response and its numerical representation within the Ad virion.

## Methods

Based on our abilities to manipulate Ad5 HVR2 and HVR5, we sought to manipulate Ad5 HVR1 in the context of HIV antigen display. More importantly, peptide incorporation within HVR1 was utilized in combination with other HVRs. In order to create a multivalent vaccine vector, we created vectors that display antigens within HVR1 and HVR2 or HVR1 and HVR5. To date this is the first report where dual antigens are displayed within one Ad hexon particle. These vectors utilize HVR1 as an incorporation site for a seven amino acid region of the HIV glycoprotein 41; in combination with a six Histidine (His6) incorporation within HVR2 or HVR5.

## Results

Our study, illustrates that these multivalent antigen vectors are viable, present HIV antigen as well as His6 within one Ad virion particle. Furthermore, mouse immunization with these vectors; demonstrate that these vectors can elicit a HIV and His6 epitope-specific humoral immune response.

## Conclusion

Our study focuses on generation of proof of concept vectors that can ultimately result in the development of multivalent vaccine vectors displaying dual antigens within the hexon of one Ad virion particle.

